# Measuring brain temperature without a thermometer

**DOI:** 10.3389/fphys.2014.00124

**Published:** 2014-03-27

**Authors:** David Papo

**Affiliations:** Computational Systems Biology Group, Center for Biomedical Technology, Universidad Politécnica de MadridMadrid, Spain

**Keywords:** fluctuation-dissipation theorem, temperature, multi-thermalization, aging, weak ergodicity breaking, cognitive neuroscience, resting state

Temperature has profound effects on a wide range of parameters of neural activity at various scales (Hodgkin and Katz, 1949). At the cell level, ionic currents, membrane potential, input resistance, action potential amplitude, duration and propagation, and synaptic transmission have all been shown to be affected by temperature variations (Hodgkin and Katz, 1949; Kullmann and Asztely, 1998; Volgushev et al., 2000a,b; Fujii et al., 2002). At mesoscopic scales of neural activity, temperature changes can steer network activity toward different functional regimes (Reig et al., 2010), affecting the duration, frequency and firing rate of activated states during slow frequency oscillations, and the ability to end these states (Compte et al., 2003). Temperature also has a substantial effect on chemical reaction rates (Swan, 1974), and affects the blood oxygen saturation level by changing haemoglobin affinity for oxygen (Guyton, 1987). Furthermore, cooling reduces metabolic processes (Esmann and Skou, 1988), and has been used to silence cortical areas to study their function (Uyeda and Fuster, 1967).

While from single cell to mesoscopic levels temperature can *directly* be measured, at the system level of non-invasive studies using electroencephalogram or functional magnetic resonance, it can only be estimated *indirectly*, using the temperature dependence of the magnetic resonance signal's frequency (Hindman, 1966; Parker et al., 1983; Kuroda et al., 1996). Furthermore, a theoretical model of brain temperature (Yablonskiy et al., 2000; Sukstankii and Yablonskiy, 2006) allows inferring from functional magnetic resonance data that functional stimulation can induce local brain temperature fluctuations of up to ±1°C with respect to resting temperature, by locally changing the balance between metabolic heat production and heat removal by blood flow.

The potential impact of temperature modulations on functional brain activity is significant. Given a temperature effect on blood oxygen saturation levels of several percent/1°C (Guyton, 1987), and an estimated average brain van't Hoff temperature coefficient Q_10_ (the factor by which a reaction rate increases for 10°C increases) of 2,3 (Swan, 1974), the observed temperature fluctuations may lead to sizeable changes in blood oxygen saturation levels and to >2% variations in chemical reaction rates.

Here we propose a way to *directly* quantify temperature from system-level brain recordings, and show how it can be used to characterize neural activity associated with cognitive function.

## Temperature as a bridge from resting to task-related brain activity

Temperature is a physical quantity that measures the mean kinetic energy of matter's particles motion. Its role is to control the energy transfer between the system and other ones to which it is thermally coupled. Temperature is an intensive property, i.e., it is shared by all the system's constituents, and independent of system size. Together with potential and other types of particle energy, it contributes to the total internal energy within a substance.

Temperature is defined as the inverse of the entropy variation △S with respect to a variation of the energy △E, at fixed volume

(1) $$-\frac{1}{T}=\frac{\partial S}{\partial E}{|}_{V,N}$$

The inverse temperature β = 1/*T is*, in essence, the cost, in entropy, of buying energy from the rest of the world (Sethna, 2006). At low temperatures, the system has few excited states and is relatively ordered; energy changes ΔE lead to large variations in the number of excited states, quantified by ΔS. High temperature corresponds to low sensitivity of entropy to variations in energy: the system is excited and disordered (Sornette, 2004).

A *bona fide* temperature ought to reflect heat flows and *thermalization*, i.e., how fluctuations relax to states in which the values of macroscopic quantities are stationary, universal with respect to differing initial conditions, and predictable (Cugliandolo et al., 1997a).

The notion of temperature is intimately related to that of *equilibrium*. Operationally, equilibrium is defined by the *zeroth law* of thermodynamics, which states that if two systems are in thermal equilibrium with a third one, they must be in thermal equilibrium with each other. The zeroth law allows using thermal equilibrium as an equivalence relationship on the set of thermally equilibrated systems, inducing a partition into subsets in mutual equilibrium. Temperature maps these subsets onto real numbers, with ordering and continuity properties.

Thermometers can be used to establish whether two systems will remain in thermal equilibrium when brought in contact. Thus, provided an appropriate thermometer can be devised, temperature can be used as a macroscopic collective variable describing the system, through which value its different subparts can be sorted.

### Thermometers and the fluctuation–dissipation theorem

A thermometer is a device, e.g. an oscillator, which when coupled to a given observable *X*, feels both its fluctuations in the absence of perturbations, measured by the two-time autocorrelation function *C_X_*(*t*, *t*′) = 〈 *X*(*t*)*X*(*t*′)〉, and the result of its own action on the system, proportional to the response function *R_X_*(*t* − *t*′), i.e., how *X* responds at time *t* to a small perturbation at time *t*' (Kurchan, 2005).

For a system at equilibrium, these two opposing effects give the correct energy, i.e., the one predicted by *equipartition theorem*, for every thermometer and observable, only if correlations and responses associated with any observable are proportional

(2) $$T=\frac{\partial C_{X}(t,\;t^{\prime })/\partial t}{R_{X}(t-t^{\prime })}=C_{X}(t,\;t^{\prime })/\chi (t,\;t^{\prime })$$

where χ (*t*, *t*′) = ∫^*t*^_*t*′_*R_X_*(*t*, τ)*d*τ is the integrated response.

The fluctuation–dissipation theorem (FDT) ensures that, for a system at equilibrium, the temperature *T* of the bath with which the system is in equilibrium is the ratio between the response to an external field conjugate to some observable and the corresponding autocorrelation function in the unperturbed system (Kubo, 1966).

In terms of brain activity, the FDT would say that stimulus-evoked brain responses can be understood through a suitable observation of the correlation of brain fluctuations at rest (Papo, 2013). Temperature represents a quantitative relationship between generic properties of ongoing brain activity and neural activity associated with cognitive function.

If the observable is the local signal energy, as is typically the case in functional imaging or electrophysiological studies, *T* quantifies the relation between energy fluctuations and the heat capacity *C_V_*:

(3) $$T^{2}\propto \frac{{\langle E-\langle E\rangle \rangle }^{2}}{C_{V}}$$

Insofar as *C_V_* measures the number of states accessible per temperature unit (DeDeo and Krakauer, 2012), temperature regulates the rate at which the system makes microstates available as a function of fluctuations in energy levels, consistent with Equation (1).

#### Fluctuation–dissipation far from equilibrium

Brain fluctuations generically show properties typical of non-equilibrium systems. The relaxation time is considerably slower than exponential (Linkenkaer-Hansen et al., 2001; Buiatti et al., 2007; Ciuciu et al., 2012; Zilber et al., 2012). Brain activity is *weakly non-ergodic* (Bianco et al., 2007), i.e., all possible states remain accessible, but some require exceedingly long times to visit (Bouchaud, 1992), and undergoes *aging* (Barkai, 2003), i.e., contrary to equilibrium fluctuations, which are time homogeneous and for which both the correlation *C* and the response function *R* depend on τ = *t* − *t_w_* elapsed from the instant *t_w_* at which a field is applied, these quantities separately depend on both *t_w_* and *t*. Preliminary evidence suggests that brain fluctuations undergo a form of aging termed *renewal aging* (Bianco et al., 2007), the possible etiologies and phenomenologies of which are discussed in Godrèche and Luck (2001); Allegrini et al. (2003); Barkai (2003); West et al. (2008); Burov et al. (2010); Barkai et al. (2012); Lomholt et al. (2013); Schulz et al. (2013) and references therein. The importance of these studies for neuroscience is huge, not least because aging is evaluated for single realizations (rather than for group averages) and this is particularly important in a field where repetitions of the same experiment encounter intrinsic difficulties.

In the presence of complex fluctuations, the FDT does not hold in its classical form (Kubo, 1966), and an appropriate generalization needs to be found. Generalized FDTs have been proposed for aging fluctuations of various kinds (Cugliandolo et al., 1997b; Crisanti and Ritort, 2003; Pottier and Mauger, 2004), including renewal aging (Allegrini et al., 2007; Aquino et al., 2007).

Out of equilibrium, the equilibrium temperature *T* no longer completely characterizes probability distributions for the system's degrees of freedom, so that, for instance, the particles' velocity and position distributions are no longer specified. Fast fluctuations thermalize to the bath temperature *T* but slow modes do not, and the direction of heat flows is characterized by an *effective temperature T_eff_* > *T* (Kurchan, 2000). *T_eff_* is, in essence, what a thermometer responding on the time scale at which the system slowly reverts to equilibrium would measure (Cugliandolo et al., 1997a). For an aging system, a generalized FDT can be written as:

(4) $$\frac{T}{X\left(t,t_{w}\right)}=\frac{\partial C\left(t,t_{w}\right)/\partial t}{R\left(t,t_{w}\right)}$$

where *X*(*t*, *t_w_*) is the fluctuation-dissipation ratio (FDR), and the ordinary FDT is recovered for *X* = 1 (Cugliandolo et al., 1997b). The time-dependent effective temperature *T_eff_*(*t, t_w_*) ∝ *T*/*X*(*t, t_w_*) allows quantifying the distance to equilibrium, and the extent to which the FDT is violated, at a given scale of activity.

As the system *ages*, the number of dynamically accessible configurational states diminishes (Angell et al., 2000) and the corresponding *T_eff_* is higher than the equilibrium temperature *T*, whereas external stimuli, force the system out of equilibrium, *rejuvenating* it (Dupuis et al., 1999; Linkenkaer-Hansen et al., 2004). *T_eff_* counts the number of metastable states of the system in the same way as *T* reflects the number of microstates at equilibrium (Martinez and Angell, 2001).

#### Multi-thermalization and dynamic heterogeneity

In an equilibrium system, any thermometer coupled to a part of the system reads the same temperature (Kurchan, 2005). In out-of-equilibrium systems, thermalization happens at widely different timescales simultaneously, within the same region of space. Correspondingly, the brain responds with avalanches spanning a broad range of scales when driven by changing external fields (Lundstrom et al., 2008).

Each timescale may be associated with its own FDR, containing information on the process relaxation, and *T_eff_* (Jack et al., 2006). A system can be at equilibrium on one scale and out of equilibrium on another, or may even be in equilibrium but show scale-dependent properties (Cugliandolo et al., 1997b; Crisanti and Ritort, 2003). Measuring *T_eff_* at various scales allows understanding the relationship between spontaneous and stimulus-induced brain activity at each scale, and the extent to which each scale of brain activity deviates from equilibrium conditions, produces entropy etc.

Furthermore, at any given time, different regions in the brain relax at different rates. *T_eff_* can be used to estimate the degree of *dynamical heterogeneity*, i.e., of spatiotemporal fluctuations in the local dynamical behavior. This can be done by calculating the *dynamic susceptibility* χ_*T*_(*t*) = ∂ 〈*C*(*t*)〉/∂*T* (Berthier et al., 2005).

## Evaluating temperature

Functionally induced brain temperature changes and the associated spatio-temporal scales can be estimated using the model of brain temperature proposed in Yablonskiy et al. (2000); Sukstankii and Yablonskiy (2006).

For brain activity at rest, the local *steady state* temperature *T*_0_ can be estimated by

(5) $$T_{0}=T_{arterial}+\frac{\left(△H^{0}-△H_{b}\right)}{\rho_{B}\cdot C_{B}}\cdot \frac{rCMRO_{2}}{rCBF}$$

where *T_arterial_* is arterial inflow temperature, △*H*^0^ the enthalpy generated by the reaction between oxygen and glucose, △*H_b_* the energy used to release oxygen from haemoglobin, ρ_*B*_ the blood heat density, *C_B_* the blood heat capacity, *rCMRO*_2_ the regional oxygen metabolic rate, and *rCBF* the regional cerebral blood flow (Yablonskiy et al., 2000).

Functional activity changes the oxygen extraction fraction *OEF* = *rCMRO*_2_/*rCBF*. Since typically *rCBF* > *rCMRO*_2_, *T* the model predicts that local changes in temperature and in *rCBF* always have opposite sign (Sukstankii and Yablonskiy, 2006).

The model estimates in the order of a few millimeters the characteristic length △ of regions where temperature changes can be observed (Sukstankii and Yablonskiy, 2006).

Changes in global *CBF* induce a temperature dynamics with a relaxation time *t_T_* = *C_tissue_*/(*rCBF* · ρ_*B*_ · *C_B_*). Estimates of *t_T_* ~ 40–60 s (Sukstankii and Yablonskiy, 2006) indicate that for *t* < *t_T_*, below the vascular response scale, measurements are out of equilibrium, *T* is not well defined, and *T_eff_* should be estimated.

Importantly, the model provides quantitative indications on *steady state* temperature modulations, and the precision with which these can be evaluated, but says little on the fluctuations that these may undergo.

## Effective temperature in real data

*T_eff_* can be estimated empirically (Martin et al., 2001; Buisson et al., 2003; Hérisson and Ocio, 2004; Mizuno et al., 2007) using standard non-invasive recordings such as electroencephalography or functional brain imaging, respectively plotting the local electrical or BOLD signal amplitude χ (*t, t*′) against *C_X_*(*t*, *t*′), and regarding brain stimulation and more generally cognitive demands can be thought of as fields pushing brain activity away from equilibrium, so that the FDT no longer holds. For instance, χ may be elicited by trains of stimuli of measurable frequency distribution (Bianco et al., 2007).

For equilibrium systems, this would yield a straight line with slope −1/*T*. Out-of-equilibrium systems typically have a more complex, system-dependent χ − *C_X_* relationship. For instance, multiscaling and aging lead to a non-linear χ − *C_X_* plot (Crisanti and Ritort, 2003), and a corresponding spectrum of slopes.

The *T_eff_*(*t, t_w_*) dependence on *t* and *t_w_* helps determining aging properties and FDT violations (Hérisson and Ocio, 2004). The former can be studied by monitoring the time evolution of *C*(*t, t_w_*) vs. (*t*, *t_w_* + τ), and by following the evolution of the linear response to a perturbation applied at *t_w_*. Deviations from the FDT can be estimated by plotting χ (*t, t_w_*) against the correlation for fixed *t_w_*, varying *t* between *t_w_* and infinity (Cugliandolo, 2011).

The estimated *T_eff_* can then be used to quantify the whole system's dynamical heterogeneity by evaluating χ_*T*_(*t*) with an appropriate *ansatz* (Berthier et al., 2005).

Depending on the recording technique, *T_eff_* could be estimated with a temporal precision ranging from the order of the temporal window within which correlations and responses are evaluated, up to *t_T_*, in the tens of seconds range (Sukstankii and Yablonskiy, 2006), and a spatial one at least of the order of the characteristic length △, of several millimeters (Parker et al., 1983).

## Conclusions

We proposed a method to measure brain temperature from any kind of non-invasive recording, which takes into account the non-equilibrium, multiscale nature of brain activity.

Effective temperature can identify, at various spatial and temporal scales the non-equilibrium regime at which the brain is working.

Temperature can be treated not only as an *order* parameter i.e., as a collective variable describing brain activity, but also as a *control* parameter, steering it to various regimes. Intuitively, cognitive processes such as learning or reasoning may be characterized as thermally-guided searches within and modifications of a complex landscape (Sherrington, 1997, 2010).

One could observe how temperature varies during the execution of a cognitive task, and then how phase transitions may occur, using temperature as a control parameter and some other property of neural activity as the order parameter.

More generally, assessing temperature and thermal history enables both a dynamical characterization of brain activity and a complete reconstruction of its thermodynamics, affording neuroscientists a description of the object of their investigations with a sound physical basis.

## References

[B1] Allegrini P. Aquino G. Grigolini P. Palatella L. Rosa A. (2003). Generalized master equation via aging continuous-time random walks. Phys. Rev. E 68:056123 10.1103/PhysRevE.68.056123 14682862

[B2] Allegrini P. Bologna M. Grigolini P. West B. J. (2007). Fluctuation-dissipation theorem for event-dominated processes. Phys. Rev. Lett. 99:010603 10.1103/PhysRevLett.99.010603 17678145

[B3] Angell C. A. Ngai K. N. McKenna G. B. McMillan P. F. Martin S. W. (2000). Relaxation in glassforming liquids and amorphous solids. J. Appl. Phys. 88, 3113–3157 10.1063/1.128603519234111

[B4] Aquino G. Grigolini P. West B. J. (2007). Linear response and fluctuation-dissipation theorem for non-Poissonian renewal processes. Europhys. Lett. 80:10002 10.1209/0295-5075/80/10002

[B5] Barkai E. (2003). Aging in subdiffusion generated by a deterministic dynamical system. Phys. Rev. Lett. 90:104101 10.1103/PhysRevLett.90.104101 12688996

[B6] Barkai E. Garini Y. Metzler R. (2012). Strange kinetics of single molecules in living cells. Phys. Today 65, 29–35 10.1063/PT.3.1677

[B7] Berthier L. Biroli G. Bouchaud J.-P. Cipelletti L. El Masri D. L'Hôte D. (2005). Direct experimental evidence of a growing length scale accompanying the glass transition. Science 310, 1797–1800 10.1126/science.1120714 16357256

[B8] Bianco S. Ignaccolo M. Rider M. S. Ross M. J. Winsor P. Grigolini P. (2007). Brain, music, and non-Poisson renewal processes. Phys. Rev. E 75:061911 10.1103/PhysRevE.75.061911 17677304

[B9] Bouchaud J.-P. (1992). Weak ergodicity breaking and aging in disordered systems. J. Phys. 2, 1705–1713

[B10] Buiatti M. Papo D. Baudonnière P. M. van Vreeswijk C. (2007). Feedback modulates the temporal scale-free dynamics of brain electrical activity in a hypothesis testing task. Neuroscience 146, 1400–1412 10.1016/j.neuroscience.2007.02.048 17418496

[B11] Buisson L. Ciliberto S. Garcimartín A. (2003). Intermittent origin of the large violations of the fluctuation-dissipation relations in an aging polymer glass. Europhys. Lett. 63, 603–609 10.1209/epl/i2003-00551-4

[B12] Burov S. Metzler R. Barkai E. (2010). Aging and nonergodicity beyond the Khinchin theorem. Proc. Natl. Acad. Sci. U.S.A. 107, 13228–13233 10.1073/pnas.1003693107 20624984 PMC2922158

[B13] Ciuciu P. Varoquaux G. Abry P. Sadaghiani S. Kleinschmidt A. (2012). Scale-free and multifractal time dynamics of fMRI signals during rest and task. Front. Physio. 3:186 10.3389/fphys.2012.00186 22715328 PMC3375626

[B14] Compte A. Sanchez-Vives M. V. McCormick D. A. Wang X. J. (2003). Cellular and network mechanisms of slow oscillatory activity (<1 Hz) and wave propagations in a cortical network model. J. Neurophysiol. 89, 2707–2725 10.1152/jn.00845.2002 12612051

[B15] Crisanti A. Ritort F. (2003). Violation of the fluctuation–dissipation theorem in glassy systems: basic notions and the numerical evidence. J. Phys. A Math. Gen. 36, R181–R290 10.1088/0305-4470/36/21/201

[B16] Cugliandolo L. F. (2011). The effective temperature. J. Phys. A Math. Theor. 44:483001 10.1088/1751-8113/44/48/483001

[B17] Cugliandolo L. F. Dean D. S. Kurchan J. (1997b). Fluctuation-dissipation theorems and entropy production in relaxational systems. Phys. Rev. Lett. 79, 2168–2171 10.1103/PhysRevLett.79.2168

[B18] Cugliandolo L. F. Kurchan J. Peliti L. (1997a). Energy flow, partial equilibration, and effective temperatures in systems with slow dynamics. Phys. Rev. E 55, 3898–3913 10.1103/PhysRevE.55.3898

[B19] DeDeo S. Krakauer D. C. (2012). Dynamics and processing in finite self-similar networks. J. R. Soc. Interface 9, 2131–2144 10.1098/rsif.2011.0840 22378750 PMC3405736

[B20] Dupuis V. Bert F. Hammann J. Ladieu F. Parker D. (1999). Aging, rejuvenation and memory phenomena in spin glasses. Pramana 53, 1–11

[B21] Esmann M. Skou J. C. (1988). Temperature-dependencies of various catalytic activities of membrane-bound Na^+^/K^+^-ATPase from ox brain, ox kidney and shark rectal gland and of C12E8-solubilized shark Na^+^/K^+^-ATPase. Biochim. Biophys. Acta 944, 344–350 10.1016/0005-2736(88)90504-4 2846060

[B22] Fujii S. Sasaki H. Ito K. Kaneko K. Kato H. (2002). Temperature dependence of synaptic responses in guinea pig hippocampal CA1 neurons *in vitro*. Cell. Mol. Neurobiol. 22, 379–391 10.1023/A:1021068919709 12507388 PMC11533788

[B23] Godrèche G. Luck J. M. (2001). Statistics of the occupation time of renewal processes. J. Stat. Phys. 104:489 10.1023/A:1010364003250

[B24] Guyton A. (1987). Textbook of Medical Physiology. Philadelphia: Saunders

[B25] Hérisson D. Ocio M. (2004). Fluctuation dissipation relation in an ageing spin glass. J. Magn. Magn. Mater. 272–276, 1280–1281 10.1016/j.jmmm.2003.12.1340

[B26] Hindman J. C. (1966). Proton resonance shift of water in gas and liquid states. J. Chem. Phys. 44, 4582–4592 10.1063/1.1726676

[B27] Hodgkin A. L. Katz B. (1949). The effect of temperature on the electrical activity of the giant axon of the squid. J. Physiol. 109, 240–249 15394322 10.1113/jphysiol.1949.sp004388 PMC1392577

[B28] Jack R. L. Berthier L. Garrahan J. P. (2006). Fluctuation-dissipation relations in plaquette spin systems with multi-stage relaxation. J. Stat. Mech. Theory Exp. P12005 10.1088/1742-5468/2006/12/P12005

[B29] Kubo R. (1966). The fluctuation-dissipation theorem. Rep. Progr. Phys. 29, 255–284 10.1088/0034-4885/29/1/306

[B30] Kullmann D. M. Asztely F. (1998). Extrasynaptic glutamate spillover in the hippocampus: evidence and implications. Trends Neurosci. 21, 8–14 10.1016/S0166-2236(97)01150-8 9464678

[B31] Kurchan J. (2000). Emergence of macroscopic temperatures in systems that are not thermodynamical microscopically: towards a thermodynamical description of slow granular rheology. J. Phys. 12, 6611–6617 10.1088/0953-8984/12/29/332

[B32] Kurchan J. (2005). In and out of equilibrium. Nature 433, 222–225 10.1038/nature03278 15662408

[B33] Kuroda K. Suzuki Y. Ishihara Y. Okamoto K. Suzuki Y. (1996). Temperature mapping using water proton chemical shift obtained with 3D-MRSI: feasibility *in vivo*. Magn. Reson. Med. 35, 20–29 10.1002/mrm.1910350105 8771019

[B34] Linkenkaer-Hansen K. Nikouline V. V. Palva J. M. Ilmoniemi R. (2001). Long-range temporal correlations and scaling behavior in human oscillations. J. Neurosci. 15, 1370–1377 11160408 10.1523/JNEUROSCI.21-04-01370.2001 PMC6762238

[B35] Linkenkaer-Hansen K. Nikouline V. V. Palva J. M. Kaila K. Ilmoniemi R. (2004). Stimulus-induced change in long-range temporal correlations and scaling behaviour of sensorimotor oscillations. Eur. J. Neurosci. 19, 203–211 10.1111/j.1460-9568.2004.03116.x 14750978

[B36] Lomholt M. A. Lizana L. Metzler R. Ambjörnsson T. (2013). Microscopic origin of the logarithmic time evolution of aging processes in complex systems. Phys. Rev. Lett. 110:208301 10.1103/PhysRevLett.110.208301 25167457

[B37] Lundstrom B. N. Higgs M. H. Spain W. J. Fairhall A. L. (2008). Fractional differentiation by neocortical pyramidal neurons. Nat. Neurosci. 11, 1335–1342 10.1038/nn.2212 18931665 PMC2596753

[B38] Martin P. Hudspeth A. J. Jülicher F. (2001). Comparison of a hair bundle's spontaneous oscillations with its response to mechanical stimulation reveals the underlying active process. Proc. Natl. Acad. Sci. U.S.A. 98, 14380–14385 10.1073/pnas.251530598 11724945 PMC64690

[B39] Martinez L.-M. Angell C. A. (2001). A thermodynamic connection to the fragility of glass-forming liquids. Nature 410, 663–667 10.1038/35070517 11287947

[B40] Mizuno D. Tardin C. Schmidt C. F. MacKintosh F. C. (2007). Nonequilibrium mechanics of active cytoskeletal networks. Science 315, 370–373 10.1126/science.1134404 17234946

[B41] Papo D. (2013). Why should cognitive neuroscientists study the brain's resting state? Front. Hum. Neurosci. 7:45 10.3389/fnhum.2013.00045 23431277 PMC3576622

[B42] Parker D. L. Smith V. Sheldon P. Crooks L. Fussel L. (1983). Temperature distribution measurements in two-dimensional NMR imaging. Med. Phys. 10, 321–325 10.1118/1.595307 6877179

[B43] Pottier N. Mauger A. (2004). Anomalous diffusion of a particle in an aging medium Physica A 332, 15–28 10.1016/j.physa.2003.10.03419479386

[B44] Reig R. Mattia M. Compte A. Belmonte C. Sanchez-Vives M. V. (2010). Temperature modulation of slow and fast cortical rhythms. J. Neurophysiol. 103, 1253–1261 10.1152/jn.00890.2009 20032235

[B45] Schulz J. H. P. Barkai E. Metzler R. (2013). Aging effects and population splitting in single-particle trajectory averages. Phys. Rev. Lett. 110:020602 10.1103/PhysRevLett.110.020602 23383881

[B46] Sethna J. P. (2006). Statistical Mechanics. Entropy, Order Parameters, and Complexity. Oxford: Clarendon Press

[B47] Sherrington D. (1997). Landscape paradigms in physics and biology: Introduction and overview. Physica D 107, 117–121 10.1016/S0167-2789(97)00076-6

[B48] Sherrington D. (2010). Physics and complexity. Phil. Trans. R. Soc. A 368, 1175–1189 10.1098/rsta.2009.0208 20123753 PMC3263799

[B49] Sornette D. (2004). Critical Phenomena in Natural Sciences, Chaos, Fractals, Self-Organization and Disorder: Concepts And Tools. 2nd Edn, Heidelberg: Springer Series in Synergetics

[B50] Sukstankii A. L. Yablonskiy D. A. (2006). Theoretical model of temperature regulation in the brain during changes in functional activity. Proc. Natl. Acad. Sci. U.S.A. 103, 12144–12149 10.1073/pnas.0604376103 16880401 PMC1567709

[B51] Sethna J. P. (2006). Statistical Mechanics. Entropy, Order Parameters, and Complexity. Oxford: Clarendon Press

[B52] Swan H. (1974). Thermoregulation and Bioenergetics. New York, NY: Elsevier

[B53] Uyeda A. A. Fuster J. M. (1967). Effects of cooling “association cortex” on visual evoked potentials. Psychol. Rep. 20, 377–378 10.2466/pr0.1967.20.2.377 4962989

[B54] Volgushev M. Vidyasagar T. R. Chistiakova M. Eysel U. T. (2000a). Synaptic transmission in the neocortex during reversible cooling. Neuroscience 98, 9–22 10.1016/S0306-4522(00)00109-3 10858607

[B55] Volgushev M. Vidyasagar T. R. Chistiakova M. Yousef T. Eysel U. T. (2000b). Membrane properties and spike generation in rat visual cortical cells during reversible cooling. J. Physiol. 522, 59–76 10.1111/j.1469-7793.2000.0059m.x 10618152 PMC2269736

[B56] West B. J. Geneston E. L. Grigolini P. (2008). Maximizing information exchange between complex networks, Phys. Rep. 468, 1–99 10.1016/j.physrep.2008.06.003

[B57] Yablonskiy D. A. Ackerman J. J. Raichle M. E. (2000). Coupling between changes in human brain temperature and oxidative metabolism during prolonged visual stimulation. Proc. Natl. Acad. Sci. U.S.A. 97, 7603–7608 10.1073/pnas.97.13.7603 10861022 PMC16592

[B58] Zilber N. Ciuciu P. Abry P. van Wassenhove V. (2012). Modulation of scale-free properties of brain activity in MEG, in IEEE International Symposium on Biomedical Imaging (Barcelona), 1531–1534

